# Persistence of antidepressant treatment in children and adolescents: A population-based cohort study

**DOI:** 10.1177/00048674261418458

**Published:** 2026-02-28

**Authors:** Gizat M Kassie, Jenni Ilomaki, Stephen J Wood, Jacqueline Gould, Melissa Raven, Jon N Jureidini, Luke E Grzeskowiak

**Affiliations:** 1 SAHMRI Women and Kids, South Australian Health and Medical Research Institute, Adelaide, SA, Australia; 2 College of Medicine and Public Health, Flinders University, Adelaide, SA, Australia; 3 Centre for Medicine Use and Safety, Monash University, Melbourne, VIC, Australia; 4 School of Public Health and Preventive Medicine, Monash University, Melbourne, VIC, Australia; 5 School of Psychology, University of Adelaide, Adelaide, SA, Australia; 6 Robinson Research Institute, University of Adelaide, Adelaide, SA, Australia

**Keywords:** Antidepressant, drug utilisation evaluation, adverse event, depression, children, adolescent

## Abstract

**Objective:**

To determine the prevalence and predictors of persistent antidepressant use among Australian children and adolescents.

**Methods:**

A population-based cohort study was conducted, including children and adolescents aged from 5 to 18 years who initiated an antidepressant between 2014 and 2022, using 10% randoms sample of Pharmaceutical Benefits Scheme (PBS) dispensing data. We measured persistence at 1 and 2 years after initiation, as defined by continuous supply of any antidepressant with no gaps of more than 90 days between dispensings.

**Results:**

A total of 44,366 children and adolescents initiated on antidepressants during the study period. Approximately one-quarter (23.1%) received only a single antidepressant dispensing, with a further 33.0% considered persistent users after 1 year and 19.8% considered persistent users after 2 years. Persistence at 1 year was significantly higher in females (adjusted odds ratios (aOR) 1.13 [1.09–1.18]) than males, and in concurrent users of antipsychotics (aOR 1.37 [1.22–1.54]) or psychostimulants (aOR 1.60 [1.49–1.71]) than non-users. The likelihood of persistent antidepressant use at 1 year was lower in individuals with a concession card (aOR 0.81 [0.78–0.85]) than general beneficiaries and in those who initiated with serotonin and norepinephrine reuptake inhibitors (aOR 0.60 [0.54–0.67]) or mirtazapine (aOR 0.45 [0.34–0.51]) compared with selective serotonin reuptake inhibitors. Findings were similar for persistent antidepressant use at 2 years.

**Conclusion:**

Persistent antidepressant use beyond 1 or 2 years is common among children and adolescents and shows an increasing trend over time. The reasons for and appropriateness of prolonged treatment with antidepressants in this population warrant further investigation.

## Introduction

Antidepressants are one of the most frequently dispensed classes of psychotropics to children and adolescents, with increasing utilisation over time, particularly during the COVID-19 pandemic (de Oliveira Costa et al., 2025; Wood et al., 2023). They are commonly used in the management of depression, but also in other conditions, including anxiety disorders, attention-deficit/hyperactivity disorder, autistic spectrum disorder, and obsessive-compulsive disorder (OCD) (Therapeutic Guidelines Limited, 2021). Some antidepressant classes, like tricyclic antidepressants, are also indicated for non-mental health conditions such as enuresis or neuropathic pain (Hazell, 2022). Increasing antidepressant use has coincided with increasing prevalence of depression and anxiety symptoms in children and adolescents (Madigan et al., 2023).

Antidepressant use in children and adolescents requires a careful balance of potential benefits and harms (Hetrick et al., 2021). Evidence indicates that antidepressants are associated with increased risk of suicide or self-harm by intentional overdose in this population and these show an incremental trend in Australia along with an increase in use of antidepressants nationally (Therapeutic Goods Administration, 2021; Whitely et al., 2020). Regarding the efficacy of antidepressant treatment in children and adolescents, a network meta-analysis concluded that they did not provide clinically significant benefits in reducing depressive symptoms (Hetrick et al., 2021) and no antidepressant demonstrated superiority over psychological interventions (Viswanathan et al., 2020). Evidence appears equally limited for other clinical indications (Locher et al., 2017).

Evidence regarding the efficacy and safety of antidepressants has largely been gathered from randomised controlled trials of relatively short duration (Zhang et al., 2018). In a recent Cochrane network meta-analysis of 26 randomised controlled trials assessing the use of antidepressants in the management of major depression in children and adolescents, treatment duration ranged from 6 to 12 weeks, with the majority of the trials involving just 8 weeks of treatment (Hetrick et al., 2021). This severely limits the assessment of potential longer-term outcomes resulting from prolonged antidepressant use. Observational studies, however, suggest that prolonged antidepressant use may be associated with a range of adverse outcomes, including reduction in growth rate (Weintrob et al., 2002), cardiovascular events (Jerrell and McIntyre, 2009) and incidence of type 2 diabetes (Movahed et al., 2025).

A recent comparative study of international clinical practice guidelines identified that some guidelines recommend antidepressants be continued for at least 6 months after resolution of symptoms, after which deprescribing may be considered (Yan et al., 2023). Data on the duration of antidepressant use in children and adolescents outside of clinical trials remains limited. A recent Scandinavian population-based study identified that 26% to 34% of children and adolescents aged from 5 to 17 years were persistent antidepressant users 1 year after initiation (Rasmussen et al., 2025). However, this study did not explore factors associated with persistence or how persistence has changed over time, particularly following the onset of the COVID-19 pandemic, which has had notable impacts on increasing the incidence of psychotropic use in children. The objective of our study was therefore to identify the prevalence and predictors of persistent antidepressant use in Australian children and adolescents who initiated these medications between 2014 and 2021.

## Methods

### Data source and study setting

We utilised the data from the Pharmaceutical Benefits Scheme (PBS) 10% sample dataset. The dataset has been widely used in drug utilisation studies and contains nationally representative, longitudinal, individual-level data obtained from a random sample of 10% of Australians eligible to receive medications subsidised under the PBS, with complete data records from 01 July 2012 (Australian Institute of Health and Welfare, 2024). The dataset contains information on dispensed medications and patients. The dataset does not include data on private prescriptions funded by the patient or private health insurer, inpatient prescriptions in public hospitals, or medications not listed on the PBS (e.g. agomelatine).

The PBS, as a key component of Australia’s universal health care scheme, Medicare, subsidises a wide range of medications for all Australian citizens, permanent residents and visitors from countries with reciprocal healthcare agreements. Patients are required to pay a co-payment of only up to a set threshold amount; any additional cost for medicines above which is covered by the government. A discounted co-payment threshold is available for those who hold a government health care card or pensioner concession card.

To be listed on the PBS, medicines have to first be approved by the Therapeutic Goods Administration (TGA), which assesses medicines for efficacy and safety against their licenced indications. Medicines listed on the PBS can be unrestricted or restricted to prescribing for specified indications. In Australia, only fluvoxamine and sertraline are specifically licenced for use in children and adolescents, for the management of OCD (Therapeutic Goods Administration, 2021). Prescribing for other clinical indications or other antidepressants, in children or adolescents, is considered ‘off-label’. In certain circumstances, for example, where a medicine is listed for treatment of major depressive disorders but not approved for use in children, medicines can still be accessed through the PBS for this indication, but use is considered ‘off-label’.

There are no restrictions on the clinical speciality of those prescribing antidepressants to children and adolescents, with prior analysis demonstrating that in Australia, most antidepressant prescriptions for children aged from 5 to 11 years are written by psychiatrists or paediatricians, while for adolescents 12 years or older, they are often written by general practitioners (Therapeutic Goods Administration, 2021).

### Study population, exposure and outcome measures

We conducted a population-based cohort study including children and adolescents aged from 5 to18 years (inclusive) who initiated an antidepressant for the first time between 2014 and 2022. We selected 2014 as the first year of the study, providing 18 months of lookback to July 2012 (the first date of data availability) to more accurately identify incident users.

Children and adolescents who initiated selective serotonin reuptake inhibitors (SSRI), serotonin and norepinephrine reuptake inhibitors (SNRI) or mirtazapine were selected. Medications were identified using Anatomical Therapeutic Chemical (ATC) classification codes provided by the World Health Organisation. The full list of antidepressants included on the PBS at the time of the study, with their respective ATC codes are presented in Supplemental Table 1. Tricyclic antidepressants (TCAs) and monoamine oxidase inhibitors (MAOIs) were not included for cohort selection as they are more commonly prescribed for other medical conditions (e.g. chronic neuropathic pain, migraine prophylaxis) or are not recommended as first-line treatments for mental health disorders according to Australian guidelines.

All children and adolescents who received a dispensing for antidepressants (SSRI, SNRI or mirtazapine) between 1 January 2014 and 31 December 2022, without having had any previous antidepressant dispensing in at least 18 months period, were considered initiators and were included in this study. That is, we only included data on the first documented initiation of an antidepressant medication for each child or adolescent. The date of the first identified antidepressant supply was termed the index date. Records were complete until December 2024; therefore, we followed up with all individuals for two full years after the index date.

Persistence with using any antidepressant at 1 and 2 years after initiation was determined for each child or adolescent. Given dose of dispensed medicines is not recorded in the PBS dataset, antidepressants were considered discontinued if no further dispensing of any antidepressant within 90 days of the last supply was identified. In such cases, discontinuation dates were conservatively assigned to be on the 30th day from the date of the last dispensing, or to the date of death if death occurred before this day. We allowed 90 days window between supplies before discontinuation was assumed to account for individuals who might have surplus medications on hand from previous supplies and for lower doses used in children, where one dispensing may last longer due to practices like taking half a tablet. Children and adolescents who did not discontinue antidepressants, whether they switched between antidepressants or not, were recorded as being persistent at the end of the follow-up periods.

We identified concurrent use of psychotropic medicines as defined by a minimum of two dispensings of anxiolytics, antipsychotics, psychostimulants or sedatives/hypnotics within 6 months before and after the date of antidepressant initiation. That is, at least one dispensing in the 6 months before antidepressant initiation and at least one dispensing in the 6 months after antidepressant initiation. The list of psychotropic medicines in the PBS with their ATC codes is available in Supplemental Table 1.

### Statistical analyses

Analyses were stratified by age group at initiation (5–11 years and 12–18 years) as management approaches and antidepressant use differ between these age groups (Therapeutic Guidelines Limited; National Institute for Health Care Excellence, 2019). Numbers and percentages of individuals persisting with antidepressants after 1 and 2 years were also presented, stratified by age group. We assessed the trend in the proportion of children and adolescents persistently using antidepressants for 1 or 2 years among initiators by calendar year using a linear regression model. Multivariable logistic regression was used to determine the odds of persistence for 1 and 2 years or single dispensing of antidepressants by patient characteristic variables. Adjusted odds ratios (aORs) with 95% confidence intervals (95% CI) were calculated, including sex, initiation year, concession card status, initiating antidepressant class and concurrent psychotropic medicine use in the model. Models were also run separately to examine whether persistence differed by the specific initiating antidepressant, adjusting for all other covariates (sex, initiation year, concession card status, and concurrent psychotropic use). Kaplan–Meier survival curves were used to present persistence by age group, with the number of days each child or adolescent persisted on antidepressant treatment calculated as the difference between the discontinuation and the index dates.

Sensitivity analyses were conducted excluding children and adolescents who received only a single antidepressant dispensing, given the possibility that a portion of these children or adolescents were dispensed medications but never initiated them. In a separate sensitivity analysis, we also visually explored patterns of persistence up to 5 years following antidepressant initiation, using a Kaplan–Meier survival curve, with censoring applied to those with less than 5 years of follow-up data.

All analyses were conducted using the statistical software package SAS version 9.4 (SAS Institute Inc., Cary, NC, USA) and the Kaplan–Meier survival curves were generated using Stata MP 17 (Stata, College Station, Texas).

## Results

### Cohort characteristics

A total of 44,366 children and adolescents (14.0% aged between 5 and 11; 86.0% aged between 12 and 18) initiated an antidepressant between January 2014 and August 2021 (Table 1). A higher proportion of incident antidepressant users aged from 12 to 18 years were female (64.7%) compared with those aged from 5 to11 years (37.5%). The most commonly initiated antidepressant class overall was SSRIs (91.8%), with fluoxetine, sertraline and escitalopram being the most frequent antidepressant types, constituting 48.0%, 20.2% and 14.5% of all the dispensings, respectively. A higher proportion of children from the 5- to 11-year-old age group (95.7%) were dispensed SSRIs than in the 12- to 18-year-old age group (91.2%). A higher proportion of children from 5 to 11 years old concurrently used psychotropic medications (29.9%) than in adolescents aged from 12 to 18 years (10.3%), with psychostimulants being the most frequently used psychotropics.

**Table 1.** Baseline characteristics of children and adolescents who initiated an antidepressant in Australia between 2014 and 2022.

Characteristic variables	All (*N* = 44,366), *N* (%)	5–11 years (*N* = 6220), *N* (%)	12–18 years (*N* = 38,146), *N* (%)
**Female sex**	27,011 (60.9%)	2330 (37.5%)	24,681 (64.7%)
**Initiation year**			
2014	3814 (8.6%)	475 (7.6%)	3339 (8.8%)
2015	4089 (9.2%)	542 (8.7%)	3547 (9.3%)
2016	4153 (9.4%)	588 (9.5%)	3565 (9.3%)
2017	4439 (10%)	624 (10.0%)	3815 (10.0%)
2018	4831 (10.9%)	724 (11.6%)	4107 (10.8%)
2019	4888 (11.0%)	739 (11.9%)	4149 (10.9%)
2020	5809 (13.1%)	812 (13.1%)	4997 (13.1%)
2021	6237 (14.1%)	834 (13.4%)	5403 (14.2%)
2022	6106 (13.8%)	882 (14.2%)	5224 (13.7%)
**Concession card holder**	16,812 (37.9%)	3305 (53.1%)	13,507 (35.4%)
**Initiating antidepressant**			
SSRI	40,736 (91.8%)	5953 (95.7%)	34,783 (91.2%)
Fluoxetine	21,301 (48.0%)	3956 (63.6%)	17,345 (45.5%)
Sertraline	8977 (20.2%)	1086 (17.5%)	7891 (20.7%)
Escitalopram	6427 (14.5%)	309 (5.0%)	6118 (16.0%)
Fluvoxamine	2256 (5.1%)	484 (7.8%)	1772 (4.6%)
Citalopram	1162 (2.6%)	84 (1.4%)	1078 (2.8%)
Paroxetine	613 (1.4%)	34 (0.5%)	579 (1.5%)
SNRI	1946 (4.4%)	155 (2.5%)	1791 (4.7%)
Desvenlafaxine	853 (1.9%)	71 (1.1%)	782 (2.1%)
Venlafaxine	652 (1.5%)	49 (0.8%)	603 (1.6%)
Duloxetine	441 (1.0%)	35 (0.6%)	406 (1.1%)
Mirtazapine	1684 (3.8%)	112 (1.8%)	1572 (4.1%)
**Concurrent use of psychotropic medicine**			
Any psychotropic medicine	5799 (13.1%)	1861 (29.9%)	3938 (10.3%)
Psychostimulants	3752 (8.5%)	1512 (24.3%)	2240 (5.9%)
Antipsychotics	1361 (3.1%)	467 (7.5%)	894 (2.3%)
Anxiolytics	668 (1.5%)	32 (0.5%)	636 (1.7%)
Sedative-hypnotics	276 (0.6%)	12 (0.2%)	264 (0.7%)

Abbreviations: SSRI, selective serotonin reuptake inhibitors; SNRI, selective norepinephrine reuptake inhibitors.

### Persistence with antidepressants

**Figure 1.**
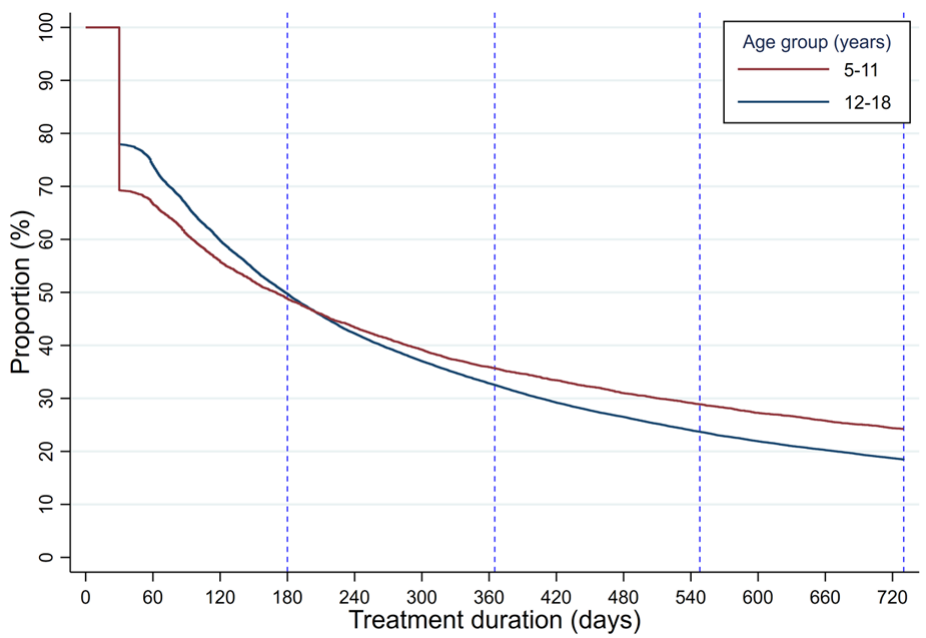
Kaplan–Meier curves indicating persistence with antidepressants over 2 years of follow-up among Australian children and adolescents aged from 5 to 18 years at antidepressant initiation, stratified by age group.

The proportion of children and adolescents considered persistent antidepressant users over the 2-year follow-up is depicted in the Kaplan–Meier curve (Figure 1). Initially, the rate of antidepressant cessation appeared higher in those aged from 5 to 11 years compared with those aged from 12 to 18 years; no difference between groups was observable at 180 days (6 months). After this time point, a greater proportion from 5 to 11 year olds were considered persistent users than those aged from 12 to 18 years.

Overall, 33.0% of those initiating antidepressants were considered persistent users after 1 year, which declined to 19.8% at 2 years. A higher proportion of children aged from 5 to 11 years persistently used antidepressants for one (35.7%) or two (24.3%) years compared with those aged from12 to 18 years (32.6% and 18.5%, respectively). In the sensitivity analysis, when children and adolescents with only a single dispensing of antidepressants were excluded from the cohort, overall persistence at 1 and 2 years was 42.9% and 25.1%, respectively. A higher proportion of children aged from 5 to 11 years persistently used antidepressants for one (51.3%) or two (34.9%) years compared with those aged from 12 to 18 years (41.7% and 23.7%, respectively).

One-year persistence was more likely among females overall (aOR 1.13 [1.09–1.18]), with this association strongest in between12 and 18 age group (aOR 1.17 [1.12–1.23]) (Table 2). A significant linear trend was evident for increasing likelihood of persistence with more recent calendar year of treatment initiation (*p* < 0.001). Among adolescents aged from 12 to 18 years, 1-year persistence was significantly associated with commencement of an antidepressant in 2018, 2019, 2020, 2021 or 2022 compared with initiations in 2014. Commonwealth healthcare concession card holders were less likely to persist for 1 year (aOR 0.81 [0.78–0.85]) than general beneficiaries. Persistence was lower in those who started with SNRIs (aOR 0.60 [0.54–0.67]) or mirtazapine (aOR 0.45 [0.40–0.51]) compared with SSRIs. Concurrent dispensing of antipsychotics (aOR 1.37 [1.23–1.54]) or psychostimulants (aOR 1.60 [1.49–1.71]) was associated with a higher likelihood of persisting with antidepressants for 1 year compared to non-users of these medicines. The factors associated with persistence at 2 years remained similar (Table 3). When stratified by individual antidepressant type, persistence at 1 and 2 years was consistently higher for those initiated on fluvoxamine compared with fluoxetine. In contrast, it was consistently lower for those initiated on other antidepressants, compared with fluoxetine (Supplemental Table 4). In a sensitivity analysis, we explored patterns of persistence up to 5 years following treatment initiation, as reflected in Supplemental Figure 1. A steady reduction in persistent use was evident across both age groups until 5 years, with approximately 4.6% of individuals identified as persistent users at 5 years.

**Table 2.** Persistence with antidepressants in Australian children and adolescents at 1 year after initiation, by age group.

Variable	All		5–11 years		12–18 years	
	*N* (%)	aOR (95% CI)	*N* (%)	aOR (95% CI)	*N* (%)	aOR (95% CI)
**Sex**						
Male	5469 (31.5%)	Reference	1379 (35.4%)	Reference	4090 (30.4%)	Reference
Female	9182 (34.0%)	**1.13 (1.09**–**1.18)**	843 (36.2%)	1.04 (0.94–1.17)	8339 (33.8%)	**1.17 (1.12**–**1.23)**
**Initiation year**						
2014	1106 (29.0%)	Reference	154 (32.4%)		952 (28.5%)	Reference
2015	1164 (28.5%)	0.98 (0.89–1.08)	173 (31.9%)	0.94 (0.72–1.23)	991 (27.9%)	0.98 (0.89–1.09)
2016	1247 (30.0%)	1.03 (0.94–1.14)	195 (33.2%)	0.98 (0.76–1.28)	1052 (29.5%)	1.04 (0.94–1.15)
2017	1353 (30.5%)	1.04 (0.95–1.15)	199 (31.9%)	0.92 (0.71–1.19)	1154 (30.2%)	1.06 (0.96–1.18)
2018	1574 (32.6%)	**1.14 (1.04–1.25)**	255 (35.2%)	1.04 (0.81–1.33)	1319 (32.1%)	**1.15 (1.04–1.28)**
2019	1695 (34.7%)	**1.25 (1.14–1.37)**	280 (37.9%)	1.18 (0.92–1.51)	1415 (34.1%)	**1.26 (1.14–1.39)**
2020	2055 (35.4%)	**1.27 (1.16–1.39)**	308 (37.9%)	1.15 (0.90–1.47)	1747 (35.0%)	**1.29 (1.17–1.42)**
2021	2226 (35.7%)	**1.27 (1.16–1.39)**	323 (38.7%)	1.18 (0.92–1.50)	1903 (35.2%)	**1.28 (1.17–1.41)**
2022	2231 (36.5%)	**1.31 (1.20–1.43)**	335 (38.0%)	1.14 (0.90–1.45)	1896 (36.3%)	**1.33 (1.21–1.46)**
**Concession card status**						
No concession card	9595 (34.8%)	Reference	1058 (36.3%)	Reference	8537 (34.6%)	Reference
Concession card holder	5056 (30.1%)	**0.81 (0.78–0.85)**	1164 (35.2%)	0.96 (0.86–1.07)	3892 (28.8%)	**0.78 (0.74–0.81)**
**Initiating antidepressant class**						
SSRI^a^	13,908 (34.1%)	Reference	2201 (37.0%)	Reference	11,707 (33.7%)	Reference
SNRI^b^	436 (22.4%)	**0.60 (0.54–0.67)**	6 (3.9%)	**0.07 (0.03–0.16)**	430 (24.0%)	**0.67 (0.60–0.75)**
Mirtazapine	307 (18.2%)	**0.45 (0.40–0.51)**	15 (13.4%)	**0.28 (0.16–0.48)**	292 (18.6%)	**0.47 (0.42–0.54)**
**Concurrent psychotropic use (Reference** **=** **No)**						
Psychostimulants	1577 (42.0%)	**1.60 (1.49–1.71)**	589 (39.0%)	**1.16 (1.03–1.32)**	988 (44.1%)	**1.80 (1.65–1.97)**
Antipsychotics	525 (38.6%)	**1.37 (1.23–1.54)**	183 (39.2%)	1.19 (0.98–1.46)	342 (38.3%)	**1.43 (1.24–1.64)**
Anxiolytics	196 (29.3%)	0.90 (0.76–1.06)	8 (25.0%)	0.70 (0.31–1.60)	188 (29.6%)	0.92 (0.77–1.09)
Sedative-hypnotics	NR	NA	NR	NA	66 (25.0%)	0.76 (0.57–1.01)

Percentages were calculated using the number of antidepressant initiators within each category as denominators; Adjusted odds ratios (aOR) were calculated, including all variables in the multivariate logistic regression model. Abbreviations: SSRI, selective serotonin reuptake inhibitors; SNRI, selective norepinephrine reuptake inhibitors; NR, not reportable; NA, not applicable.

a SSRIs include fluoxetine, sertraline, escitalopram, fluvoxamine, citalopram, and paroxetine.

b SNRIs include desvenlafaxine, venlafaxine and duloxetine.

**Table 3.** Persistence with antidepressants in Australian children and adolescents at 2 years after initiation, by age group.

Variable	All		5–11 years		12–18 years	
	*N* (%)	aOR (95% CI)	*N* (%)	aOR (95% CI)	*N* (%)	aOR (95% CI)
**Sex**						
Male	3249 (18.7%)	Reference	938 (24.1%)	Reference	2311 (17.2%)	Reference
Female	5311 (19.7%)	**1.10 (1.05–1.16)**	571 (24.5%)	1.04 (0.92–1.18)	4740 (19.2%)	**1.17 (1.11–1.24)**
**Initiation year**						
2014	614 (16.1%)	Reference	99 (20.8%)		515 (15.4%)	Reference
2015	669 (16.4%)	1.02 (0.91–1.16)	114 (21.0%)	0.98 (0.72–1.33)	555 (15.6%)	1.03 (0.90–1.17)
2016	690 (16.6%)	1.02 (0.91–1.15)	133 (22.6%)	1.06 (0.79–1.43)	557 (15.6%)	1.01 (0.88–1.15)
2017	798 (18.0%)	1.11 (0.99–1.25)	141 (22.6%)	1.05 (0.79–1.41)	657 (17.2%)	1.12 (0.99–1.27)
2018	936 (19.4%)	**1.21 (1.08–1.36)**	173 (23.9%)	1.10 (0.83–1.46)	763 (18.6%)	**1.22 (1.08–1.39)**
2019	996 (20.4%)	**1.29 (1.16–1.44)**	176 (23.8%)	1.11 (0.84–1.47)	820 (19.8%)	**1.32 (1.17–1.49)**
2020	1216 (20.9%)	**1.32 (1.19–1.47)**	209 (25.7%)	1.21 (0.92–1.59)	1007 (20.2%)	**1.33 (1.19–1.5)**
2021	1297 (20.8%)	**1.30 (1.17–1.44)**	236 (28.3%)	1.37 (1.04–1.79)	1061 (19.6%)	**1.28 (1.14–1.43)**
2022	1344 (22.0%)	**1.39 (1.25–1.54)**	228 (25.9%)	1.21 (0.92–1.58)	1116 (21.4%)	**1.41 (1.26–1.58)**
**Concession card status**						
No	5491 (19.9%)	Reference	704 (24.2%)	Reference	4787 (19.4%)	Reference
Yes	3069 (18.3%)	**0.89 (0.85–0.94)**	805 (24.4%)	1.01 (0.90–1.14)	2264 (16.8%)	**0.84 (0.80–0.89)**
**Initiating antidepressant class**						
SSRI^a^	8141 (20.0%)	Reference	1495 (25.1%)	Reference	6646 (19.1%)	Reference
SNRI^b^	242 (12.4%)	**0.61 (0.53–0.70)**	3 (1.9%)	**0.06 (0.02–0.20)**	239 (13.3%)	**0.70 (0.61–0.81)**
Mirtazapine	177 (10.5%)	**0.49 (0.42–0.57)**	11 (9.8%)	**0.34 (0.18–0.64)**	166 (10.6%)	**0.52 (0.44–0.62)**
**Concurrent psychotropic use (Reference** **=** **No)**						
Psychostimulants	1050 (28.0%)	**1.73 (1.60–1.87)**	415 (27.4%)	**1.20 (1.05–1.37)**	635 (28.3%)	**1.90 (1.72–2.10)**
Antipsychotics	347 (25.5%)	**1.47 (1.29–1.67)**	131 (28.1%)	**1.24 (1.00–1.54)**	216 (24.2%)	**1.51 (1.29–1.77)**
Anxiolytics	121 (18.1%)	0.99 (0.81–1.21)	5 (15.6%)	0.68 (0.26–1.78)	116 (18.2%)	1.04 (0.85–1.28)
Sedative-hypnotics	NR	NA	NR	NA	40 (15.2%)	0.87 (0.62–1.22)

Percentages were calculated using the number of antidepressant initiators within each category as denominators; Adjusted odds ratios (aOR) were calculated, including all variables in the multivariate logistic regression model. Other antidepressants included reboxetine and mianserin. Abbreviations: SSRI, selective serotonin reuptake inhibitors; SNRI, selective norepinephrine reuptake inhibitors; NR, not reportable; NA, not applicable.

a SSRIs include fluoxetine, sertraline, escitalopram, fluvoxamine, citalopram, and paroxetine.

b SNRIs include desvenlafaxine, venlafaxine and duloxetine.

In a sensitivity analysis, excluding those who received only a single dispensing, the factors associated with persistence with antidepressants at 1 and 2 years were found to be similar (Supplemental Tables 2 and 3).

### Single dispensing of antidepressants

Approximately one in four (23.3%) initiators received only a single antidepressant dispensing. Receiving a single antidepressant dispensing was higher in children from 5 to 11 years (30.4%) than in those from 12 to 18 years (21.9%). Concession card holders, and those initiating their treatment with SNRI, mirtazapine or other antidepressants (compared with SSRIs) were more likely to receive only one dispensing of antidepressants. On the other hand, females (compared with males) and concurrent users of psychostimulants or antipsychotics (compared with non-users of these medicines) were less likely to receive only one antidepressant dispensing (Table 4). A significant linear trend was also evident, with the likelihood of receiving a single dispensing of an antidepressant decreasing over time (*p* < 0.001).

**Table 4.** Receipt of single dispensing only among Australian children and adolescents initiated on antidepressants, by age group.

Variable	All		5–11 years		12–18 years	
	*N* (%)	aOR (95% CI)	*N* (%)	aOR (95% CI)	*N* (%)	aOR (95% CI)
**Sex**						
Male	4380 (25.2%)	Reference	1213 (31.2%)	Reference	3167 (23.5%)	Reference
Female	5863 (21.7%)	**0.84 (0.80–0.88)**	679 (29.1%)	0.89 (0.79–1.00)	5184 (21%)	0.88 (0.84–0.93)
**Initiation year**						
2014	1004 (26.3%)	Reference	154 (32.4%)	Reference	850 (25.5%)	Reference
2015	1039 (25.4%)	0.94 (0.85–1.04)	170 (31.4%)	0.99 (0.76–1.29)	869 (24.5%)	0.94 (0.84–1.05)
2016	1078 (26.0%)	1.00 (0.90–1.11)	192 (32.7%)	1.07 (0.82–1.38)	886 (24.9%)	0.99 (0.88–1.10)
2017	1132 (25.5%)	1.00 (0.90–1.10)	224 (35.9%)	1.25 (0.96–1.61)	908 (23.8%)	0.95 (0.85–1.06)
2018	1144 (23.7%)	0.91 (0.83–1.01)	228 (31.5%)	1.05 (0.82–1.35)	916 (22.3%)	**0.88 (0.79–0.98)**
2019	1150 (23.5%)	0.91 (0.82–1.00)	204 (27.6%)	0.86 (0.67–1.11)	946 (22.8%)	0.91 (0.81–1.01)
2020	1206 (20.8%)	**0.79 (0.71–0.87)**	239 (29.4%)	0.97 (0.76–1.24)	967 (19.4%)	**0.75 (0.67–0.83)**
2021	1262 (20.2%)	**0.78 (0.71–0.86)**	225 (27.0%)	0.87 (0.68–1.11)	1037 (19.2%)	**0.76 (0.68–0.84)**
2022	1228 (20.1%)	**0.78 (0.71–0.86)**	256 (29.0%)	0.96 (0.75–1.23)	972 (18.6%)	**0.74 (0.66–0.82)**
**Concession card status**						
No concession card	5845 (21.2%)	Reference	872 (29.9%)	Reference	4973 (20.2%)	Reference
Concession card holder	4398 (26.2%)	**1.28 (1.22–1.34)**	1020 (30.9%)	1.05 (0.94–1.17)	3378 (25.0%)	**1.28 (1.22–1.35)**
**Initiating antidepressant class**						
SSRI^a^	8889 (21.8%)	Reference	1744 (29.3%)	Reference	7145 (20.5%)	Reference
SNRI^b^	683 (35.1%)	**1.83 (1.66–2.02)**	93 (60.0%)	**3.48 (2.51–4.84)**	590 (32.9%)	**1.78 (1.6–1.97)**
Mirtazapine	671 (39.8%)	**2.25 (2.03–2.49)**	55 (49.1%)	**2.25 (1.54–3.27)**	616 (39.2%)	**2.37 (2.14–2.64)**
**Concurrent psychotropic use (Reference** **=** **No)**						
Psychostimulants	781 (20.8%)	**0.82 (0.75–0.89)**	403 (26.7%)	**0.80 (0.70–0.91)**	378 (16.9%)	**0.68 (0.61–0.77)**
Antipsychotics	278 (20.4%)	**0.75 (0.65–0.86)**	122 (26.1%)	**0.79 (0.63–0.98)**	156 (17.4%)	**0.67 (0.56–0.80)**
Antipsychotics Anxiolytics	152 (22.8%)	0.92 (0.77–1.11)	12 (37.5%)	1.14 (0.54–2.40)	140 (22.0%)	0.94 (0.78–1.14)
Sedative–hypnotics	78 (28.3%)	1.16 (0.89–1.51)	9 (75.0%)	**5.32 (1.38–20.41)**	69 (26.1%)	1.12 (0.85–1.48)

Percentages were calculated using the number of antidepressant initiators within each category as denominators; Adjusted odds ratios (aOR) were calculated, including all variables in the multivariate logistic regression model. Other antidepressants included reboxetine and mianserin. Abbreviations: SSRI, selective serotonin reuptake inhibitors; SNRI, selective norepinephrine reuptake inhibitors.

a SSRIs include fluoxetine, sertraline, escitalopram, fluvoxamine, citalopram, and paroxetine.

b SNRIs include desvenlafaxine, venlafaxine and duloxetine.

## Discussion

One third (33.0%) of children and adolescents who commenced on antidepressants remained persistent users after 1 year, and nearly one in four (19.8%) after 2 years. When extrapolated to the entire Australian paediatric population aged from 5 to 18 years in 2022, the proportions equate to approximately 22,310 and 13,440 children and adolescents who initiated on antidepressants in the year and remained persistent users after 1 and 2 years, respectively. The likelihood of persistent antidepressant use varied significantly according to various factors, including age, gender, calendar year, antidepressant type, being a concession card holder, and concurrent psychotropic medication use. Given uncertainties associated with prolonged antidepressant use, the appropriateness of these prescribing patterns warrants further attention.

Our observed prevalence of persistent antidepressant use at 1 year (33%) was lower than that reported in a prior study in Denmark (Pottegård et al., 2014), which found persistence exceeding 40%. This discrepancy is likely due to methodological differences, particularly the permissible gap between dispensings, with the former one allowing a 180-day gap, whereas we used a 90-day gap. Furthermore, evolving safety concerns and clinical guidelines since the Danish study may have influenced prescribing practices, contributing to lower persistence in our cohort. However, our result is comparable to another more recent study in Denmark (31%), Sweden (34%), and Norway (26%) (Rasmussen et al., 2025).

After 2 years, almost one in five children and adolescents (18%) remained persistent users, but we found no comparable studies exploring this outcome. Collectively, these findings raise questions about the potential appropriateness of prolonged antidepressant treatment and raise concerns about potential overtreatment. The optimal duration of antidepressant use depends on various factors, including treatment response, adverse effects, parent/family preference, number of previous depressive episodes and co-existing conditions (Dwyer and Bloch, 2019). Clinical practice guidelines, including Australian guidelines, recommend considering deprescribing after 6 months of continuous use with adequate response (Therapeutic Guidelines Limited; Malhi et al., 2021). These recommendations are mirrored in international guidelines (National Institute for Health Care Excellence, 2019; Yan et al., 2023). Some international guidelines recommend extended treatment for 1 year or more where individuals have had recurrent major depressive episodes in the past (Yan et al., 2023), but this is unlikely to be relevant to our cohort, given that we restricted it to new antidepressant users.

The efficacy and safety of long-term use of antidepressants in children and adolescents remain uncertain, with emerging evidence that prolonged antidepressant use may heighten the risk of adverse outcomes (Jerrell and McIntyre, 2009; Movahed et al., 2025; Weintrob et al., 2002). Extended use of antidepressants is also associated with worsening of withdrawal symptoms upon ceasing (Horowitz and Wilcock, 2022; Viswanathan et al., 2020). Findings related to high rates of persistent antidepressant use suggest a need to improve the awareness and skills of prescribers in deprescribing (Stutzman, 2021).

Consistent with a previous study in Finland that included data from 1999 to 2005 (Saastamoinen et al., 2012), our findings showed that younger children were more likely to continue antidepressant treatment for longer periods than older adolescents. In our study, children aged from 5 to 11 years demonstrated higher persistence rates, with 35.7% of initiators continuing treatment after 1 year and 24.3% after 2 years, from 12 to 18 year olds (32.6% and 18.5%, respectively). This could be associated with a higher prevalence of co-existing mental health conditions in this age group. This suggestion is supported by the higher rate of concurrent psychotropic use in the younger age group (one in three children), predominantly psychostimulants, than in the older age group (one in ten adolescents). Our findings revealed that children and adolescents with concurrent use of psychostimulants or antipsychotics exhibited significantly higher odds of persistence than those with no dispensing of these medications. Family or parental involvement in decisions regarding treatment course is another important factor to consider, which could have more impact in determining duration in younger children than in adolescents.

There were no observed differences in persistence between sexes in the younger age group. However, females had a higher likelihood of persisting with antidepressants than males in the older age group. This aligns with evidence that increasing age was associated with more depression symptoms in females, but not in males (Klaufus et al., 2024), which may contribute to longer treatment periods in females (Balzen et al., 2023). Another study has also shown that remission from depressive symptoms at 6 months, among adolescents treated with antidepressants, was less likely in females than males (Balzen et al., 2023). In addition, we found that adolescents who initiated antidepressants from 2019 to 2022 were more likely to continue their medication for 1 or 2 years than those who initiated from 2014. This could be linked to the well-documented exacerbating effects of the COVID-19 pandemic and corresponding restrictions on mental health issues in children (Madigan et al., 2023). Australian studies showed that COVID-19-related restrictions impacted physical access to non-pharmacologic mental health services in children and adolescents (Podubinski et al., 2021; Sicouri et al., 2023). Concession card holders were less likely to persist with antidepressants, which could be due to the inability to afford medications or general practitioner, paediatrician or psychiatrist consultations to obtain repeat prescriptions. Previous evidence also shows that children or adolescents whose parents had low socioeconomic status were less likely to continue with their treatments (Kalaman et al., 2023).

This study revealed that a significant proportion (23.3%) of children and adolescents received only a single dispensing of an antidepressant. We could not evaluate how many of these were cases in which antidepressants were dispensed but never taken, or cases in which antidepressants were commenced but discontinued relatively rapidly. Such discontinuation could be due to adverse effects and/or lack of treatment response.

In the case of adverse effects or lack of response with antidepressant treatment, clinical practice guidelines recommend dose changes or switching to a different antidepressant (Therapeutic Guidelines Limited; Grover and Avasthi, 2019). This study has identified factors associated with a higher likelihood of receiving only a single dispensing of antidepressants in children, such as being a concession card holder, and initiating with SNRIs, or mirtazapine (compared with SSRIs). Further research is needed to investigate the reasons underlying the single dispensing of antidepressants in children and adolescents.

### Strengths and limitations

Our study has key strengths worth noting. First, it utilised nationwide data with a large sample size, offering a comprehensive representation of antidepressant use across the country. Second, the 2-year follow-up period from antidepressant initiation allowed for a more thorough identification of persistent users.

However, some limitations should be considered when interpreting the findings. First, we could not confirm whether the dispensed antidepressants were actually taken by the patients, because we used administrative claims data. Nonetheless, this method is a closer proxy for medicine use than using prescribing data. Dates of treatment discontinuation are estimated based on the final date a medication was dispensed and may therefore differ from the true date of discontinuation. In addition, we had no data available on medical diagnoses or reasons for prescribing. We attempted to mitigate this issue by excluding certain antidepressant groups commonly prescribed for non-mental health conditions in children and not typically used as first-line initiation treatments.

Because this study used dispensing data alone, it was not possible to evaluate the use of psychotherapy (often provided by psychologists) or disease severity, which may be an important factor in the duration of antidepressant use. However, out-of-pocket expenses and psychologist shortages may restrict access to psychotherapy, especially during the COVID-19 pandemic when mental health presentations, including depression, significantly increased in Australia (John et al., 2023).

## Conclusions

This study found that persistent use of antidepressants for extended periods is common in Australian children and adolescents, indicating potential overtreatment. Higher likelihoods of persistent use were evident in female adolescents, younger children, and concurrent users of psychostimulants and antipsychotics, the underlying reasons for which may warrant closer examination. Future prospective research should explore more factors contributing to extended use of antidepressants, including utilisation of psychotherapy and other mental health treatments. Furthermore, there is a pressing need to evaluate treatment outcomes.

## Supplemental Material

sj-docx-1-anp-10.1177_00048674261418458 – Supplemental material for Persistence of antidepressant treatment in children and adolescents: A population-based cohort study Supplemental material, sj-docx-1-anp-10.1177_00048674261418458 for Persistence of antidepressant treatment in children and adolescents: A population-based cohort study by Gizat M Kassie, Jenni Ilomaki, Stephen J Wood, Jacqueline Gould, Melissa Raven, Jon N Jureidini and Luke E Grzeskowiak in Australian & New Zealand Journal of Psychiatry

sj-tif-2-anp-10.1177_00048674261418458 – Supplemental material for Persistence of antidepressant treatment in children and adolescents: A population-based cohort study Supplemental material, sj-tif-2-anp-10.1177_00048674261418458 for Persistence of antidepressant treatment in children and adolescents: A population-based cohort study by Gizat M Kassie, Jenni Ilomaki, Stephen J Wood, Jacqueline Gould, Melissa Raven, Jon N Jureidini and Luke E Grzeskowiak in Australian & New Zealand Journal of Psychiatry
