# Supplementary material for: Persistence of antidepressant treatment in children and adolescents: A population-based cohort study
Source: Aust N Z J Psychiatry. 2026 Feb 28;60(7):643–52. doi: 10.1177/00048674261418458 (PMC13291400; doi:10.1177/00048674261418458)
Supplement: sj-docx-1-anp-10.1177_00048674261418458 – Supplemental material for Persistence of antidepressant treatment in children and adolescents: A population-based cohort study [file sj-docx-1-anp-10.1177_00048674261418458.docx]

**Persistence of antidepressant treatment in children and adolescents: A population-based cohort study**

**Supplementary appendix**

**Table of contents**

[**Supplemental table 1:** Pharmaceutical Benefits Scheme item codes used to identify psychotropic medications, with the corresponding Anatomical Therapeutic Chemical 5 codes 2](#_Toc214623947)

[**Supplemental table 2:** Persistence with antidepressants among Australian children and adolescents one year after initiation (excluding those with single dispensing) 4](#_Toc214623948)

[**Supplemental table 3:** Persistence with antidepressants among Australian children and adolescents two years after initiation (excluding those with single dispensing) 5](#_Toc214623949)

[**Supplemental table 4:** Persistence with antidepressants among Australian children and adolescents, and receipt of single dispensing, stratified by initiating antidepressant 6](#_Toc214623950)

[**Supplemental figure 1:** Kaplan-Meier curves indicating persistence with antidepressants over five years of follow-up among Australian children and adolescents aged 5 to 18 years at antidepressant initiation, stratified by age group. 7](#_Toc214623951)

# **Supplemental table 1:** Pharmaceutical Benefits Scheme item codes used to identify psychotropic medications, with the corresponding Anatomical Therapeutic Chemical 5 codes

| **Medicine name** | | **PBS item code/s** | **ATC 5 Code** |
| --- | --- | --- | --- |
| **Antidepressants** | | | |
| **Selective serotonin reuptake inhibitors** | | | |
| Citalopram | | 08220P; 08702B; 08703C | N06AB04 |
| Escitalopram | | 08700X; 08701Y; 08849R; 09432K; 09433L; 10181W | N06AB10 |
| Fluoxetine | | 01434L; 08270G; 12256C | N06AB03 |
| Fluvoxamine | | 08174F; 08512B | N06AB08 |
| Paroxetine | | 02242B; 09197C | N06AB05 |
| Sertraline | | 02236Q; 02237R; 08836C; 08837D; 12299H | N06AB06 |
| **Serotonin and norepinephrine reuptake inhibitors** | | | |
| Desvenlafaxine | | 09366Y; 09367B; 10231L; 10234P; 10241B; 10245F | N06AX23 |
| Duloxetine | | 09155W; 09156X | N06AX21 |
| Reboxetine | | 08583R | N06AX18 |
| Venlafaxine | | 08301X; 08302Y; 08868R | N06AX16 |
| **Tetracyclic antidepressants** | | | |
| Mianserin | | 01627P; 01628Q | N06AX03 |
| Mirtazapine | | 08513C; 08855C; 08856D; 08857E; 08883M; 09365X | N06AX11 |
| **Tricyclic antidepressants** | | | |
| Amitriptyline | | 02417F; 02418G; 02429W | N06AA09 |
| Clomipramine | | 01561E | N06AA04 |
| Dosulepin (dothiepin) | | 01357K; 01358L | N06AA16 |
| Doxepin | | 01011F; 01012G; 01013H | N06AA12 |
| Imipramine | | 02420J; 02421K; 12113M; 12581E | N06AA02 |
| Nortriptyline | | 02522R; 02523T | N06AA10 |
| **Monoamine oxidase inhibitors** | | | |
| Phenelzine | | 02856H | N06AF03 |
| Tranylcypromine | | 02444P | N06AF04 |
| Moclobemide | | 01900B; 08003F | N06AG02 |
| **Mood stabilizer** | | | |
| Lithium carbonate | | 03059B; 08290H | N06AN01 |
| **Antipsychotics** | | | |
| Amisulpride | | 08594H; 08595J; 08596K; 08736T | N05AL05 |
| Aripiprazole | | 08717T; 08718W; 08719X; 08720Y; 10219W; 10224D | N05AX12 |
| Asenapine | | 05140M; 05141N | N05AH05 |
| Brexpiprazole | | 11184P; 11188W; 11189X; 11190Y | N05AX16 |
| Chlorpromazine | | 01195X; 01196Y; 01197B; 01199D; 01201F | N05AA01 |
| Clozapine | | 05626D; 05627E; 05628F; 05629G; 06102E; 06417R; 06418T; 10288L; 10289M; 10302F; 10358E | N05AH02 |
| Flupentixol decanoate | | 02255Q; 02257T | N05AF01 |
| Haloperidol | | 02761H; 02763K; 02767P; 02768Q; 02770T; 12519X; 02765M; 02766N | N05AD01 |
| Lurasidone | | 10526B; 10529E | N05AE05 |
| Olanzapine | | 01024X; 01037N; 01042W; 03381Y; 03382B; 03384D; 03385E; 08170B; 08185T; 08186W; 08187X; 08433W; 08434X; 08952E; 08953F; 09294E; 09295F; 09303P | N05AH03 |
| Paliperidone | | 05100K; 05102M; 05103N; 05107T; 05109X; 09140C; 09141D; 09142E; 11066K; 11072R; 11085K; 11094X | N05AX13 |
| Periciazine | | 03052P; 03053Q; 11413Q | N05AC01 |
| Quetiapine | | 05458G; 08456C; 08457D; 08458E; 08580N; 09202H; 09203J; 09204K; 09205L | N05AH04 |
| Risperidoney | | 01842Y; 01846E; 03169T; 03170W; 03171X; 03172Y; 08100H; 08780D; 08781E; 08782F; 08787L; 08788M; 08789N; 08790P; 08792R; 08794W; 08869T; 08870W; 09075P; 09076Q; 09079W; 09080X; 09293D; 11869Q; 11872W; 11873X; 11874Y; 11877D; 11879F; 11881H; 11882J | N05AX08 |
| Trifluoperazine | | 02185B; 02186C; 02386N | N05AB06 |
| Ziprasidone | | 09070J; 09071K; 09072L; 09073M | N05AE04 |
| Zuclopenthixol decanoate | | 08097E | N05AF05 |
| **Sedative/hypnotics** | | | |
| Temazepam | | 02089Y; 02088X; 05221T | N05CD07 |
| Nitrazepam | | 05359C; 05360D; 05189D; 02723H; 02732T | N05CD02 |
| **Psychostimulants** | | | |
| Atomoxetine | 09092M; 09093N; 09094P; 09095Q; 09096R; 09289X; 09290Y | | N06BA09 |
| Dexamfetamine | 01165H; | | N06BA02 |
| Lisdexamfetamine | 11884L; 11897E; 11898F; 10474G; 10486X; 10492F | | N06BA12 |
| Methylphenidate | 12116Q; 08839F; 03440C; 02432B; 02276T; 02280B; 02283E; 02387P; 02388Q; 02172H | | N06BA04 |
| Modafinil | 08816B | | N06BA07 |
| Armodafinil | 10912H; 10919Q; 10922W | | N06BA13 |
| **Anxiolytics** | | | |
| Diazepam | 02558P; 03161J; 03162K; 05071X; 05072Y; 05355W; 05356X; 05357Y; 05358B | | N05BA01 |
| Alprazolam | 02130D; 02131E; 02132F; 08118G; 11186R; 11187T; 11205R | | N05BA12 |
| Oxazepam | 03132W; 03133X; 05193H | | N05BA04 |

**Note:** BPS, Pharmaceutical Benefits Scheme; ATC, Anatomical Therapeutic Chemical

# **Supplemental table 2:** Persistence with antidepressants among Australian children and adolescents one year after initiation (excluding those with single dispensing)

| **Variable** | **All** | | **5-11 years** | | **12-18 years** | |
| --- | --- | --- | --- | --- | --- | --- |
|  | **N (%)** | **aOR (95% CI)** | **N (%)** | **aOR** **(95% CI)** | **N (%)** | **aOR (95% CI)** |
| **Sex** |  |  |  |  |  |  |
| Male | 5469 (42.2%) | Reference | 1379 (51.5%) | Reference | 4090 (39.7%) | Reference |
| Female | 9182 (43.4%) | **1.08 (1.03-1.13)** | 843 (51.1%) | 0.98 (0.87-1.12) | 8339 (42.8%) | **1.15 (1.09-1.2)** |
| **Initiation year** |  |  |  |  |  |  |
| 2014 | 1106 (39.4%) | Reference | 154 (48%) | Reference | 952 (38.2%) | Reference |
| 2015 | 1164 (38.2%) | 0.96 (0.86-1.06) | 173 (46.5%) | 0.94 (0.69-1.27) | 991 (37.0%) | 0.96 (0.86-1.07) |
| 2016 | 1247 (40.6%) | 1.04 (0.94-1.16) | 195 (49.2%) | 1.02 (0.76-1.38) | 1052 (39.3%) | 1.04 (0.93-1.16) |
| 2017 | 1353 (40.9%) | 1.05 (0.94-1.16) | 199 (49.8%) | 1.04 (0.77-1.40) | 1154 (39.7%) | 1.05 (0.94-1.17) |
| 2018 | 1574 (42.7%) | **1.12 (1.02-1.24)** | 255 (51.4%) | 1.10 (0.83-1.46) | 1319 (41.3%) | **1.12 (1.01-1.25)** |
| 2019 | 1695 (45.3%) | **1.25 (1.13-1.38)** | 280 (52.3%) | 1.13 (0.86-1.50) | 1415 (44.2%) | **1.26 (1.13-1.40)** |
| 2020 | 2055 (44.6%) | **1.20 (1.09-1.33)** | 308 (53.8%) | 1.20 (0.91-1.58) | 1747 (43.3%) | **1.20 (1.08-1.33)** |
| 2021 | 2226 (44.7%) | **1.20 (1.09-1.32)** | 323 (53%) | 1.14 (0.87-1.50) | 1903 (43.6%) | **1.20 (1.08-1.33)** |
| 2022 | 2231 (45.7%) | **1.24 (1.13-1.36)** | 335 (53.5%) | 1.16 (0.89-1.53) | 1896 (44.6%) | **1.24 (1.12-1.37)** |
| C**oncession card status** |  |  |  |  |  |  |
| No concession card | 9595 (44.2%) | Reference | 1058 (51.8%) | Reference | 8537 (43.4%) | Reference |
| Concession card holder | 5056 (40.7%) | **0.86 (0.83-0.91)** | 1164 (50.9%) | 0.99 (0.87-1.12) | 3892 (38.4%) | **0.82 (0.78-0.86)** |
| **Initiating antidepressant** |  |  |  |  |  |  |
| SSRI^%^ | 13908 (43.7%) | Reference | 2201 (52.3%) | Reference | 11707 (42.4%) | Reference |
| SNRI^§^ | 436 (34.5%) | **0.72 (0.64-0.81)** | 6 (9.7%) | **0.10 (0.04-0.24)** | 430 (35.8%) | **0.80 (0.71-0.91)** |
| Mirtazapine | 307 (30.3%) | **0.58 (0.51-0.66)** | 15 (26.3%) | **0.34 (0.19-0.62)** | 292 (30.5%) | **0.62 (0.54-0.72)** |
| **Concurrent psychotropic use (reference = No)** |  |  |  |  |  |  |
| Psychostimulants | 1577 (53.1%) | **1.61 (1.49-1.74)** | 589 (53.1%) | 1.06 (0.92-1.22) | 988 (53.1%) | **1.73 (1.57-1.91)** |
| Antipsychotics | 525 (48.5%) | **1.29 (1.14-1.46)** | 183 (53%) | 1.09 (0.87-1.37) | 342 (46.3%) | **1.30 (1.12-1.51)** |
| Anxiolytics | 196 (38.0%) | 0.86 (0.71-1.02) | 8 (40.0%) | 0.69 (0.28-1.72) | 188 (37.9%) | 0.89 (0.74-1.07) |
| Sedative-hypnotics | NA | 0.75 (0.56-1.01) | NR | NA | 66 (33.8%) | 0.76 (0.56-1.02) |

**Note:** Percentages were calculated using the number of antidepressant initiators within each category as denominators; Adjusted odds ratios (aOR) were calculated including all variables in the multivariate logistic regression model. ^%^ SSRIs include fluoxetine, sertraline, escitalopram, fluvoxamine, citalopram, and paroxetine. ^§^ SNRIs include desvenlafaxine, venlafaxine and duloxetine. Other antidepressants included reboxetine and mianserin. Abbreviations: SSRI, selective serotonin reuptake inhibitors; SNRI, selective norepinephrine reuptake inhibitors; NR, not reportable; NA, not applicable.

# **Supplemental table 3:** Persistence with antidepressants among Australian children and adolescents two years after initiation (excluding those with single dispensing)

| **Variable** | **Total** |  | **5-11 years** | | **12-18 years** | |
| --- | --- | --- | --- | --- | --- | --- |
|  | **N (%)** | **aOR (95% CI)** | **N (%)** | **aOR** **(95% CI)** | **N (%)** | **aOR (95% CI)** |
| **Sex** |  |  |  |  |  |  |
| Male | 3249 (25%) | Reference | 938 (35%) | Reference | 2311 (22.4%) | Reference |
| Female | 5311 (25.1%) | 1.05 (1.00-1.11) | 571 (34.6%) | 0.99 (0.87-1.13) | 4740 (24.3%) | **1.14 (1.08-1.21)** |
| **Initiation year** |  |  |  |  |  |  |
| 2014 | 614 (21.9%) | Reference | 99 (30.8%) | Reference | 515 (20.7%) | Reference |
| 2015 | 669 (21.9%) | 1.01 (0.89-1.14) | 114 (30.6%) | 0.98 (0.71-1.36) | 555 (20.7%) | 1.01 (0.88-1.16) |
| 2016 | 690 (22.4%) | 1.02 (0.91-1.16) | 133 (33.6%) | 1.11 (0.81-1.52) | 557 (20.8%) | 1.00 (0.88-1.15) |
| 2017 | 798 (24.1%) | 1.12 (0.99-1.26) | 141 (35.3%) | 1.19 (0.87-1.63) | 657 (22.6%) | 1.11 (0.97-1.26) |
| 2018 | 936 (25.4%) | **1.20 (1.07-1.35)** | 173 (34.9%) | 1.17 (0.86-1.58) | 763 (23.9%) | 1.19 (1.05-1.36) |
| 2019 | 996 (26.6%) | **1.28 (1.14-1.43)** | 176 (32.9%) | 1.06 (0.78-1.43) | 820 (25.6%) | **1.31 (1.15-1.48)** |
| 2020 | 1216 (26.4%) | **1.26 (1.12-1.41)** | 209 (36.5%) | 1.24 (0.93-1.67) | 1007 (25%) | **1.25 (1.11-1.41)** |
| 2021 | 1297 (26.1%) | **1.23 (1.10-1.37)** | 236 (38.8%) | **1.35 (1.01-1.8)** | 1061 (24.3%) | **1.20 (1.06-1.35)** |
| 2022 | 1344 (27.6%) | **1.32 (1.18-1.47)** | 228 (36.4%) | 1.23 (0.92-1.64) | 1116 (26.2%) | **1.32 (1.17-1.49)** |
| **Concession card status** |  |  |  |  |  |  |
| No concession card | 5491 (25.3%) | Reference | 704 (34.5%) | Reference | 4787 (24.3%) | Reference |
| Concession card holder | 3069 (24.7%) | 0.95 (0.91-1.01) | 805 (35.2%) | 1.05 (0.92-1.19) | 2264 (22.4%) | **0.9 (0.85-0.95)** |
| **Initiating antidepressant class** |  |  |  |  |  |  |
| SSRI^%^ | 8141 (25.6%) | Reference | 1495 (35.5%) | Reference | 6646 (24%) | Reference |
| SNRI^§^ | 242 (19.2%) | **0.73 (0.64-0.85)** | 3 (4.8%) | **0.10 (0.03-0.31)** | 239 (19.9%) | **0.83 (0.72-0.96)** |
| Mirtazapine | 177 (17.5%) | **0.63 (0.53-0.74)** | 11 (19.3%) | **0.45 (0.23-0.87)** | 166 (17.4%) | **0.69 (0.58-0.81)** |
| **Concurrent psychotropic use (reference = No)** |  |  |  |  |  |  |
| Psychostimulants | 1050 (35.3%) | **1.71 (1.58-1.86)** | 415 (37.4%) | 1.11 (0.96-1.28) | 635 (34.1%) | **1.81 (1.63-2.00)** |
| Antipsychotics | 347 (32.0%) | **1.39 (1.21-1.58)** | 131 (38.0%) | 1.16 (0.91-1.46) | 216 (29.3%) | **1.40 (1.18-1.64)** |
| Anxiolytics | 121 (23.4%) | 0.97 (0.79-1.19) | 5 (25.0%) | 0.67 (0.24-1.87) | 116 (23.4%) | 1.03 (0.84-1.28) |
| Sedative-hypnotics | NA | NA | NR | NA | 40 (20.5%) | 0.89 (0.63-1.26) |

**Note:** Percentages were calculated using the number of antidepressant initiators within each category as denominators; Adjusted odds ratios (aOR) were calculated including all variables in the multivariate logistic regression model. ^%^ SSRIs include fluoxetine, sertraline, escitalopram, fluvoxamine, citalopram, and paroxetine. ^§^ SNRIs include desvenlafaxine, venlafaxine and duloxetine. Other antidepressants included reboxetine and mianserin. Abbreviations: SSRI, selective serotonin reuptake inhibitors; SNRI, selective norepinephrine reuptake inhibitors; NR, not reportable; NA, not applicable.

# **Supplemental table 4:** Persistence with antidepressants among Australian children and adolescents, and receipt of single dispensing, stratified by initiating antidepressant

| **Variable** | **Total** |  | **5-11 years** | | **12-18 years** | |
| --- | --- | --- | --- | --- | --- | --- |
|  | **N (%)** | **aOR (95% CI)** | **N (%)** | **aOR** **(95% CI)** | **N (%)** | **aOR (95% CI)** |
| **One year persistence** |  |  |  |  |  |  |
| Fluoxetine | 7662 (36.0%) | Reference | 1504 (38%) | Reference | 6158 (35.5%) | Reference |
| Sertraline | 2978 (33.2%) | **0.89 (0.84-0.93)** | 394 (36.3%) | **0.92 (0.80-1.06)** | 2584 (32.7%) | **0.89 (0.84-0.94)** |
| Escitalopram | 1914 (29.8%) | **0.76 (0.72-0.81)** | 67 (21.7%) | **0.46 (0.35-0.60)** | 1847 (30.2%) | **0.80 (0.75-0.85)** |
| Fluvoxamine | 910 (40.3%) | **1.25 (1.14-1.37)** | 218 (45.0%) | **1.37 (1.13-1.66)** | 692 (39.1%) | **1.21 (1.10-1.34)** |
| Mirtazapine | 307 (18.2%) | **0.42 (0.37-0.48)** | 15 (13.4%) | **0.27 (0.15-0.46)** | 292 (18.6%) | **0.44 (0.39-0.50)** |
| Citalopram | 291 (25.0%) | **0.62 (0.54-0.71)** | 18 (21.4%) | **0.46 (0.27-0.78)** | 273 (25.3%) | **0.65 (0.56-0.74)** |
| Desvenlafaxine | NR | NA | NR | NA | 195 (24.9%) | **0.67 (0.57-0.79)** |
| Venlafaxine | NR | NA | NR | NA | 137 (22.7%) | **0.57 (0.47-0.69)** |
| Paroxetine | NR | NA | NR | NA | 153 (26.4%) | **0.70 (0.58-0.85)** |
| Duloxetine | NR | NA | NR | NA | 98 (24.1%) | **0.62 (0.49-0.78)** |
| **Two years persistence** |  |  |  |  |  |  |
| Fluoxetine | 4447 (20.9%) | Reference | 1022 (25.8%) | Reference | 3425 (19.7%) | Reference |
| Sertraline | 1780 (19.8%) | 0.95 (0.89-1.01) | 260 (23.9%) | 0.9 (0.77-1.06) | 1520 (19.3%) | 0.98 (0.92-1.05) |
| Escitalopram | 1106 (17.2%) | **0.81 (0.76-0.87)** | 46 (14.9%) | **0.51 (0.37-0.71)** | 1060 (17.3%) | **0.87 (0.81-0.94)** |
| Fluvoxamine | 563 (25.0%) | **1.30 (1.17-1.44)** | 156 (32.2%) | **1.41 (1.14-1.73)** | 407 (23%) | **1.25 (1.11-1.41)** |
| Mirtazapine | 177 (10.5%) | **0.47 (0.40-0.55)** | 11 (9.8%) | **0.33 (0.18-0.62)** | 166 (10.6%) | **0.51 (0.43-0.60)** |
| Citalopram | 161 (13.9%) | **0.65 (0.55-0.77)** | 11 (13.1%) | **0.46 (0.24-0.87)** | 150 (13.9%) | **0.70 (0.58-0.83)** |
| Desvenlafaxine | NR | NA | NR | NA | 107 (13.7%) | **0.72 (0.58-0.88)** |
| Venlafaxine | NR | NA | NR | NA | 83 (13.8%) | **0.69 (0.55-0.87)** |
| Paroxetine | NR | NA | NR | NA | 84 (14.5%) | **0.74 (0.59-0.94)** |
| Duloxetine | NR | NA | NR | NA | 49 (12.1%) | **0.60 (0.44-0.81)** |
| **Receipt of single dispensing** |  |  |  |  |  |  |
| Fluoxetine | 4251 (20.0%) | Reference | 1106 (28%) | Reference | 3145 (18.1%) | Reference |
| Sertraline | 2103 (23.4%) | **1.23 (1.16-1.31)** | 329 (30.3%) | **1.13 (0.98-1.31)** | 1774 (22.5%) | **1.30 (1.22-1.39)** |
| Escitalopram | 1559 (24.3%) | **1.30 (1.21-1.39)** | 146 (47.2%) | **2.30 (1.82-2.91)** | 1413 (23.1%) | **1.34 (1.25-1.44)** |
| Fluvoxamine | 458 (20.3%) | 0.98 (0.88-1.10) | 107 (22.1%) | **0.72 (0.58-0.91)** | 351 (19.8%) | 1.08 (0.95-1.22) |
| Mirtazapine | 671 (39.8%) | **2.51 (2.27-2.79)** | 55 (49.1%) | **2.4 (1.64-3.50)** | 616 (39.2%) | **2.75 (2.46-3.07)** |
| Citalopram | 304 (26.2%) | **1.38 (1.21-1.59)** | 38 (45.2%) | **2.07 (1.33-3.20)** | 266 (24.7%) | **1.41 (1.22-1.63)** |
| Desvenlafaxine | 278 (32.6%) | **1.82 (1.56-2.11)** | 45 (63.4%) | **4.35 (2.66-7.11)** | 233 (29.8%) | **1.76 (1.50-2.06)** |
| Venlafaxine | 239 (36.7%) | **2.23 (1.90-2.63)** | 29 (59.2%) | **3.63 (2.04-6.46)** | 210 (34.8%) | **2.29 (1.93-2.72)** |
| Paroxetine | 214 (34.9%) | **2.06 (1.74-2.45)** | 18 (52.9%) | **2.76 (1.40-5.45)** | 196 (33.9%) | **2.18 (1.83-2.60)** |
| Duloxetine | 166 (37.6%) | **2.31 (1.90-2.81)** | 19 (54.3%) | **2.91 (1.49-5.7)** | 147 (36.2%) | **2.42 (1.96-2.97)** |

**Note:** Percentages were calculated using the number of antidepressant initiators within each category as denominators; Adjusted odds ratios (aOR) were calculated including all variables in the multivariate logistic regression model (sex, age group, initiation year, concession card status, concurrent psychotropic a medicine use). Abbreviations: NR, not reportable; NA, not applicable.

**
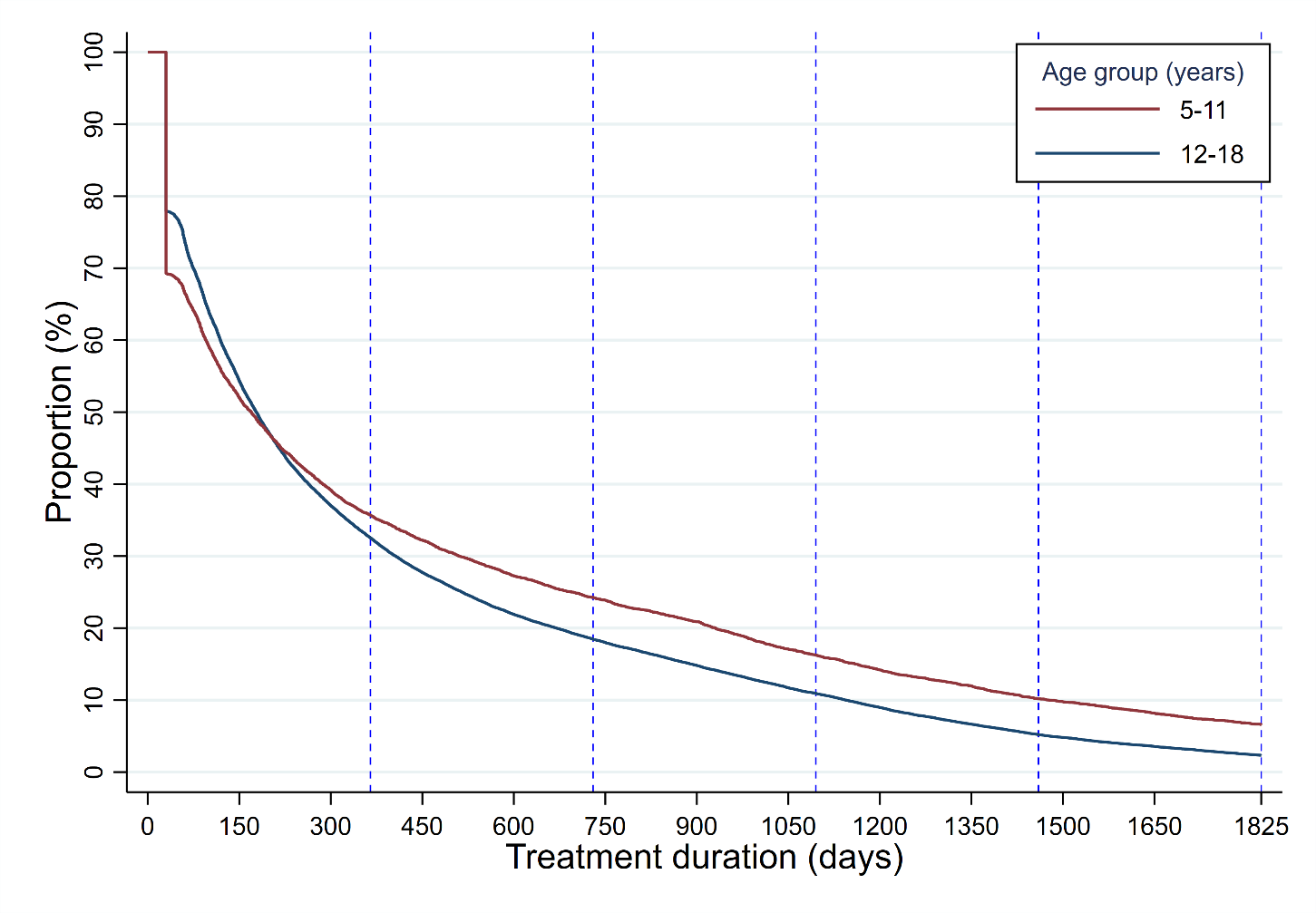
**

# **Supplemental figure 1:** Kaplan-Meier curves indicating persistence with antidepressants over five years of follow-up among Australian children and adolescents aged 5 to 18 years at antidepressant initiation, stratified by age group.
