# Supplementary figures and images for: Persistence of antidepressant treatment in children and adolescents: A population-based cohort study
Source: Aust N Z J Psychiatry. 2026 Feb 28;60(7):643–52. doi: 10.1177/00048674261418458 (PMC13291400; doi:10.1177/00048674261418458)

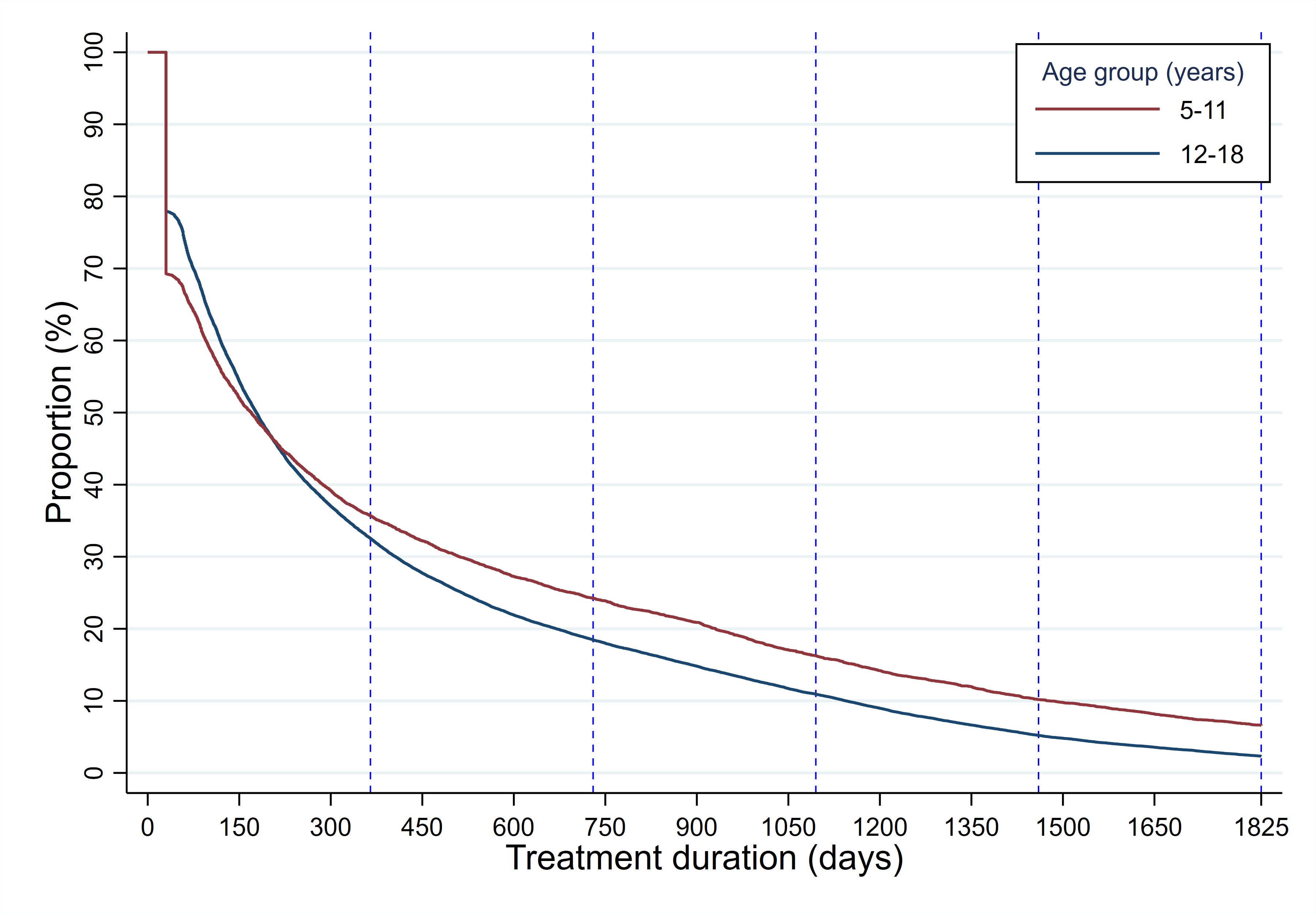

Supplement: sj-tif-2-anp-10.1177_00048674261418458 – Supplemental material for Persistence of antidepressant treatment in children and adolescents: A population-based cohort study [file sj-tif-2-anp-10.1177_00048674261418458.tif]
